# Ten-year clinical outcomes of everolimus- and biolimus-eluting coronary stents vs. everolimus-eluting bioresorbable vascular scaffolds—insights from the EVERBIO-2 trial

**DOI:** 10.3389/fcvm.2024.1426348

**Published:** 2024-09-10

**Authors:** Samir Bengueddache, Malica Cook, Sonja Lehmann, Diego Arroyo, Mario Togni, Serban Puricel, Stephane Cook

**Affiliations:** Department of Cardiology, Fribourg University and Hospital, Fribourg, Switzerland

**Keywords:** percutaneous coronary intervention (PCI), drug-eluting stent (DES), bioresorbable vascular scaffold (BVS), randomized clinical trial, coronary artery disease

## Abstract

**Background:**

Bioresorbable vascular scaffolds (BVSs) have been developed as a potential solution to mitigate late complications associated with drug-eluting metallic stents (DESs) in percutaneous coronary intervention for coronary artery disease. While numerous studies have compared BVSs to DESs, none have assessed clinical outcomes beyond 5 years.

**Objectives:**

This study aimed to compare the 10-year clinical outcomes of patients treated with BVSs vs. DESs.

**Methods:**

The EverBio-2 trial (Comparison of Everolimus- and Biolimus-Eluting Coronary Stents with Everolimus-Eluting Bioresorbable Vascular Scaffold) is a single-center, assessor-blinded, randomized controlled trial that enrolled 240 patients allocated in a 1:1:1 ratio to receive BVSs, everolimus-eluting stents, or biolimus-eluting stents (BESs). Clinical follow-up was scheduled for 10 years.

**Results:**

Clinical follow-up was completed in 222 patients (93%) at the 10-year mark. The rate of device-oriented composite events (DOCE) was 28% in the DES group and 29% in the BVS group (*p* = 0.72) at 10 years. Similarly, the rate of patient-oriented composite events (POCE) was 55% in the DES group and 49% in the BVS group (*p* = 0.43) at 10 years. Notably, the rate of myocardial infarction (MI) within the target vessel was 5% in the BVS group and 0% in the BES group (*p* = 0.04), while the rate of any MI was 10% in the BVS group and 2% in the BES group (*p* = 0.04). In addition, the rate of Academic Research Consortium (ARC) possible stent thrombosis was 3% in the BVS group and 0% in the DES group (*p* = 0.04).

**Conclusions:**

Over 10 years, the rates of clinical DOCE and POCE were similar between the BVS and DES groups but individual outcomes of stent thrombosis were higher (3%) in the BVS group compared to the DES group.

**Clinical Trial Registration:**

ClinicalTrials.gov, identifier (NCT01711931).

## Introduction

In interventional cardiology, bioresorbable vascular scaffolds (BVSs) have gained attention as an alternative to drug-eluting stents (DESs) since the first fully bioresorbable scaffold was described by Igaki-Tamai in 1998. The Absorb BVS was the first bioresorbable scaffold with drug-elution in 2006 (1). While DESs are the standard for percutaneous coronary intervention (PCI), BVSs were aimed to reduce late-occurring complications such as neoatherosclerosis and late stent thrombosis (2). Initially deemed safe for simple lesions (3), subsequent RCTs found the Absorb BVS comparable to DES at 1 year but divergent outcomes at 3 years (4–7), leading to early termination of one study due to higher device thrombosis rates at 2 years (8). Two meta-analyses confirmed increased scaffold thrombosis (9, 10). In early 2017, the European Society of Cardiology - European Association of Percutaneous Cardiovascular Interventions task force advised against routine BVS use, limiting it primarily to research (11). The most extensively studied BVS was withdrawn in September 2017, followed by an FDA warning (12). Ongoing monitoring of patients who received this BVS confirmed increased complications, while research has explored tailored stenting techniques, such as the “Predilatation, Sizing and Postdilatation” technique, in high-risk restenosis patients. These studies have yielded mixed results, from BVS non-inferiority to higher device thrombosis risks (13–17). Although the use of BVSs has been completely discontinued, over a million patients have been treated, and follow-up of these patients is necessary. In this regard, the current study presents the 10-year clinical follow-up of patients included in the EverBio-2 study.

## Methods

The EverBio-2 trial (Comparison of Everolimus- and Biolimus-Eluting Coronary Stents with Everolimus-Eluting Bioresorbable Vascular Scaffold) is a single-center, assessor-blinded, randomized controlled trial in which 240 patients were randomly allocated (1:1:1) to the BVS, everolimus-eluting stent (EES), or biolimus-eluting stent (BES) groups and was conducted at University & Hospital Fribourg. The methods of the trial have previously been reported (18–21). Clinical follow-up was initially planned at 3, 6, 9, and 12 months and at 2, 5, and 10 years. Angiographic follow-up was scheduled for 9 months and 5 years. Clinical follow-up was conducted through clinic visits, phone calls, or correspondence. The primary endpoint was late lumen loss at 9 months. Clinical endpoints included Academic Research Consortium (ARC)-defined composites: device-oriented clinical events (DOCEs), which is a composite of cardiac death, target-vessel myocardial infarction (TV-MI), and target lesion revascularization (TLR); and patient-oriented clinical events (POCEs), which is a composite of all-cause death, any MI, and any revascularization. In addition, device thrombosis and target-vessel revascularization (TVR) were also assessed (22). Blinded assessors adjudicated angiographic and clinical outcomes. A complete list of the endpoints can be found elsewhere. All patients provided written, informed consent for participation and the study protocol conforms to the ethical guidelines of the 1975 Declaration of Helsinki. The trial is registered in ClinicalTrials.gov, number (NCT01711931).

### Studied devices

The Absorb BVS (Abbott Vascular) has a poly-dl-lactide coating that releases everolimus and is completely degraded via the Krebs cycle. The scaffold has 150-μm struts. The Promus Element stent (Boston Scientific, Marlborough, USA) is a platinum chromium alloy with everolimus (100 μg/cm^2^) applied in a durable polymer. The Biomatrix Flex stent (Biosensors Europe SA, Morges, Switzerland) is stainless steel (strut thickness of 112 μm) with an abluminal biodegradable polymer layer (20 μm) that elutes biolimus.

### Statistical analysis

Categorical variables are reported as counts and percentages and continuous variables are reported as means and standard deviations. Categorical variables were compared using Fisher's exact test. Continuous variables were analyzed using Student’s *t*-test or the Wilcoxon rank-sum test according to their distribution. Survival analysis was performed using Kaplan–Meier curves and the log-rank test. Patients who received metallic stents were compared to patients treated with BVSs. To fully disclose the results, *post hoc* inferential statistics were performed comparing the distinct metallic DES individually to the BVS. All statistical analyses were performed using dedicated software (Stata version 18, StataCorp LP, College Station, TX, USA) at a two-tailed significance level of *α* = 0.05.

## Results

### Baseline patient characteristics

**Figure 1:**
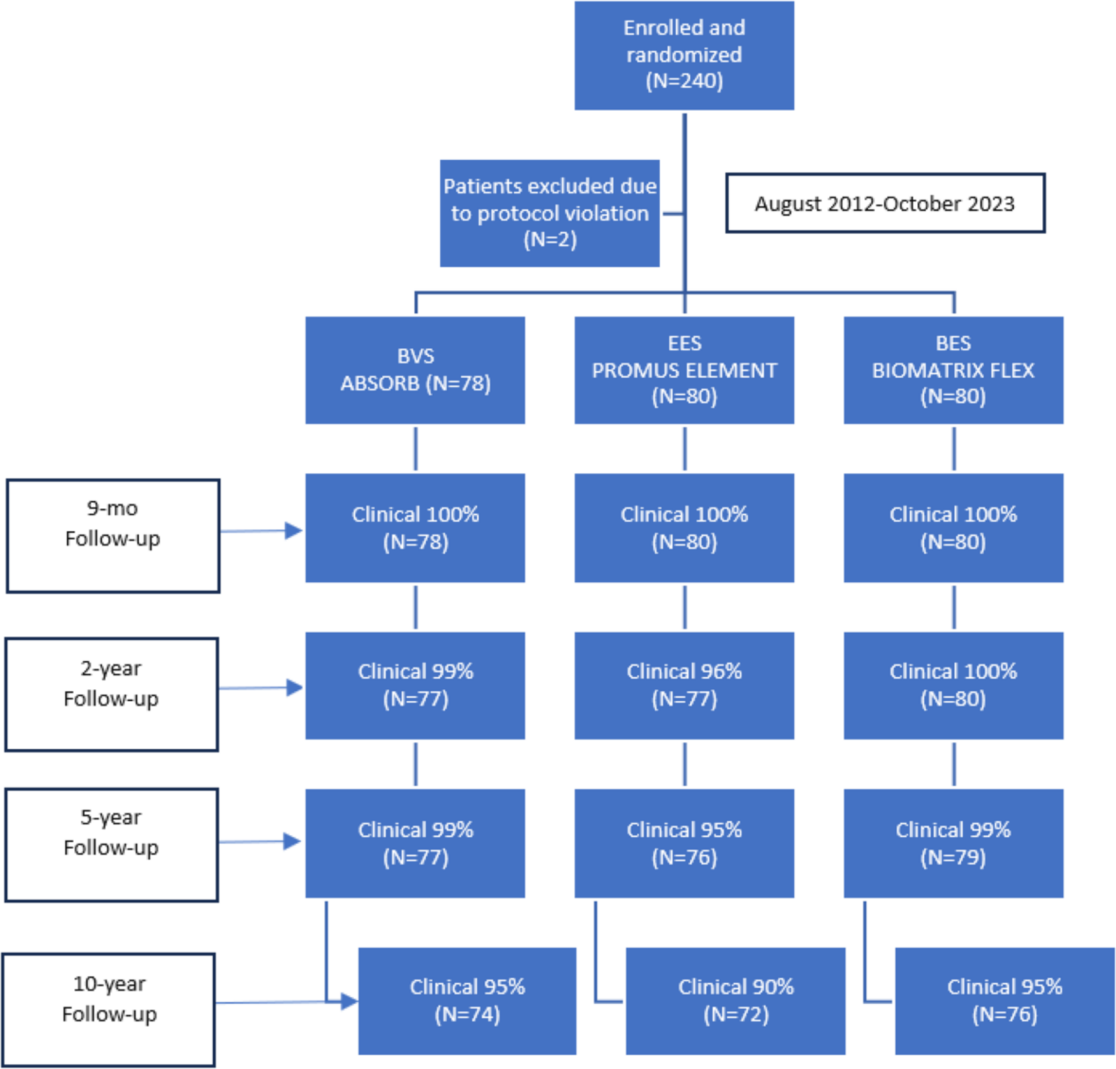
Patient flow chart.

The flowchart up to 10 years follow-up is depicted in Figure 1. Baseline patient (Table 1) and angiographic and procedural (Table 2) characteristics were well balanced between the treatment arms. From the initial cohort of 240 patients enrolled in the study in 2012, 222 patients (93%) were available for clinical follow-up at 10 years. This represented 95% (*n* = 76) of patients in the EES group, 90% (*n* = 72) in the BES group, and 95% (*n* = 74) in the BVS group.

**Table 1:** Patient baseline characteristics.

					*p-*value		
	BVS (*n* = 74)	EES/BES (*n* = 148)	EES (*n* = 72)	BES (*n* = 76)	BVS vs. EES/BES	BVS vs. EES	BVS vs. BES
Male	59 (80)	117 (79)	57 (79)	60 (79)	1.00	1.00	1.00
Age, years	74 (11)	73 (11)	74 (11)	73 (10)	0.86	0.85	0.62
Hypertension	40 (54)	92 (62)	45 (62)	47 (62)	0.25	0.32	0.41
Diabetes	14 (19)	37 (25)	12 (17)	25 (33)	0.40	0.83	0.06
Non-insulin dependent	14 (19)	27 (18)	7 (10)	20 (26)	1.00	0.16	0.33
Smoking	26 (35)	51 (34)	27 (38)	24 (32)	1.00	0.87	0.73
Dyslipidemia	42 (57)	95 (64)	45 (62)	50 (66)	0.31	0.50	0.32
Family history of CAD	20 (27)	41 (28)	19 (26)	22 (29)	1.00	1.00	0.86
Previous PCI	23 (31)	45 (30)	23 (32)	22 (29)	1.00	1.00	0.86
Previous CABG	6 (8)	24 (16)	10 (14)	14 (18)	0.14	0.30	0.09
Previous MI	10 (14)	27 (18)	12 (17)	15 (20)	0.45	0.65	0.38
Indication for index procedure					0.99	0.67	0.55
Silent ischemia	10 (14)	21 (14)	6 (8)	15 (20)			
Stable angina	35 (47)	68 (46)	42 (58)	26 (34)			
Unstable angina	5 (7)	10 (7)	3 (4)	7 (9)			
NSTEMI	16 (22)	35 (24)	15 (21)	20 (26)			
STEMI	8 (11)	14 (9)	6 (8)	8 (11)			
LVEF,%	59 ± 19	57 ± 17	58 ± 10	55 ± 20	0.16	0.48	0.09

EES, everolimus-eluting stent; BES, biolimus-eluting stent; BVS, bioresorbable vascular scaffold; CAD, coronary artery disease; PCI, percutaneous coronary intervention; CABG, coronary artery bypass grafting; NSTEMI, non-ST-elevation myocardial infarction; STEMI, ST-elevation myocardial infarction; LVEF, left ventricular ejection fraction.

Values are *n* (%), mean ± SD, or median (interquartile range).

**Table 2:** Baseline angiographic characteristics.

					*p-*value		
	BVS (*n* = 74)	EES/BES (*n* = 148)	EES (*n* = 72)	BES (*n* = 76)	BVS vs. EES/BES	BVS vs. EES	BVS vs. BES
Diseased vessels per patient	1.9 ± 0.8	1.9 ± 0.8	1.9 ± 0.8	1.9 ± 0.8	0.67	0.77	0.66
Treated vessels per patient	1.1 ± 0.3	1.2 ± 0.4	1.2 ± 0.4	1.1 ± 0.4	0.53	0.47	0.70
Lesions per patient	2.1 ± 1.4	2.1 ± 1.2	2.1 ± 1.3	2.1 ± 1.2	0.97	0.96	0.91
Treated lesions per patient	1.3 ± 0.5	1.4 ± 0.5	1.5 ± 0.8	1.3 ± 0.6	0.29	0.10	0.85
Total Lesions	*n* = 92	*n* = 209	*n* = 101	*n* = 108			
Target coronary artery							
LM	0 (0)	2 (1)	1 (1)	1 (1)	1.00	1.00	1.00
LAD	44 (48)	76 (36)	40 (40)	36 (33)	0.07	0.31	0.04*
LCX	24 (26)	46 (22)	21 (21)	25 (23)	0.46	0.40	0.74
RCA	22 (24)	85 (41)	38 (38)	47 (44)	0.01*	0.04*	0.01*
Arterial graft	0 (0)	3 (1)	2 (2)	1 (1)	0.56	0.50	1.00
Vein graft	4 (4)	10 (5)	3 (3)	7 (6)	1.00	0.71	0.55
Lesion complexity							
A	20 (22)	56 (27)	25 (25)	31 (29)	0.35	0.73	0.33
B1	46 (50)	85 (41)	41 (41)	44 (41)	0.13	0.20	0.20
B2	11 (12)	33 (16)	16 (16)	17 (16)	0.39	0.54	0.54
C	15 (16)	35 (17)	19 (19)	16 (15)	0.92	0.71	0.85
Baseline TIMI flow per lesion							
TIMI 0	0 (0)	3 (1)	2 (2)	1 (1)	0.25	0.50	1.00
TIMI 1	0 (0)	0 (0)	0 (0)	0 (0)	—	—	—
TIMI 2	1 (1)	1 (1)	1 (1)	0 (0)	0.55	1.00	0.46
TIMI 3	91 (99)	205 (98)	98 (97)	107 (99)	0.61	0.62	1.00
TIMI flow post-intervention per lesion							
TIMI 0	0 (0)	0 (0)	0 (0)	0 (0)	—	—	—
TIMI 1	0 (0)	0 (0)	0 (0)	0 (0)	—	—	—
TIMI 2	0 (0)	1 (0)	1 (1)	0 (0)	0.51	1.00	1.00
TIMI 3	92 (100)	208 (100)	100 (99)	108 (100)	0.51	1.00	1.00
Restenotic lesion	1 (1)	4 (2)	1 (1)	3 (3)	1.00	1.00	0.63
Chronic total occlusion	1 (1)	12 (6)	7 (7)	5 (5)	0.12	0.07	0.22
Thrombus aspiration	7 (8)	12 (6)	5 (5)	7 (6)	0.61	0.56	0.79
Number of stent per lesion	1.2 ± 0.5	1.2 ± 0.6	1.3 ± 0.7	1.1 ± 0.4	0.87	0.08	0.05*
Lesion length, mm	23 ± 9	21 ± 12	22 ± 14	19 ± 10	0.11	0.61	0.01*
Maximum pressure per lesion, atm	14 ± 2.8	14 ± 3.0	15 ± 2.8	14 ± 3.1	0.14	0.01*	0.92
Overlapping stents per lesion	14 (15)	35 (17)	23 (23)	12 (11)	0.87	0.20	0.41
Direct stenting per lesion	2 (2)	38 (18)	16 (16)	22 (20)	<0.001*	<0.001*	<0.001*
Post-dilatation per lesion	31 (34)	63 (30)	30 (30)	33 (31)	0.59	0.64	0.65
Baseline MLD, mm	2.4 ± 0.5	2.2 ± 0.5	2.2 ± 0.5	2.2 ± 0.6	0.01*	<0.001*	0.02*

BES, biolimus-eluting stent; BVS, bioresorbable vascular scaffold; EES, everolimus-eluting stent; LAD, left anterior descending; LCX, left circumflex artery; LM, left main coronary artery; RCA, right coronary artery; MLD, minimum lumen diameter.

Values are mean ± SD or *n* (%).

*p-value < 0.05.

### Clinical outcomes at 10 years

Clinical adverse events are detailed in Table 3.

**Table 3:** Outcomes at 10 years.

					*p-*value		
	BVS (*n* = 78)	EES/BES (*n* = 160)	EES (*n* = 80)	BES (*n* = 80)	BVS vs. EES/BES	BVS vs. EES	BVS vs. BES
Device-oriented composite	23 (29)	44 (28)	22 (28)	22 (28)	0.72	0.90	0.62
Cardiac death	7 (9)	14 (9)	5 (6)	9 (11)	0.94	0.65	0.59
MI of target vessel	4 (5)	2 (1)	2 (2)	0 (0)	0.08	0.41	0.04*
TLR	17 (22)	33 (21)	18 (22)	15 (19)	0.82	0.86	0.54
Patient-oriented composite	38 (49)	88 (55)	44 (55)	44 (55)	0.43	0.30	0.76
All-cause mortality	11 (14)	29 (18)	15 (19)	14 (18)	0.34	0.33	0.52
Any MI	8 (10)	10 (6)	8 (10)	2 (2)	0.28	0.99	0.04*
Any revascularization	29 (37)	69 (43)	35 (44)	34 (42)	0.50	0.38	0.78
Possible stent thrombosis	2 (3)	0 (0)	0 (0)	0 (0)	0.04*	0.16	0.15
Probable/definite stent thrombosis	0 (0)	0 (0)	0 (0)	0 (0)	—	—	—

EES, everolimus-eluting stent; BES, biolimus-eluting stent; BVS, bioresorbable vascular scaffold; MI, myocardial infarction; TLR; target lesion revascularization.

Values are *n* (%); *p*-values are derived from log-rank test.

*p-value < 0.05.

ARC-defined DOCEs occurred in 23 (29%) patients treated with a BVS, 22 (28%) patients in the EES group, and 22 (28%) patients in the BES group. There was no significant difference in the DOCE rates between the metallic DES (*n* = 44, 28%) and a BVS (*n* = 23, 29%; *p* = 0.72). Regarding individual endpoints, the rate of MI within the target vessel was significantly higher in the BVS group (*n* = 4, 5%) compared to the BES group (0, 0%; *p* = 0.04), with a trend toward significance when compared to the DES group (2, 1%; *p* = 0.08).

There was no significant difference in the POCEs between the DES (*n* = 88, 55%) and BVS groups (*n* = 38, 49%; *p* = 0.43). However, the rate of MI was significantly higher in the BVS group (*n* = 8, 10%) compared to the BES group (2, 2%; *p* = 0.04).

Possible stent thrombosis according to ARC definitions (22) occurred significantly more frequently in the BVS group (*n* = 2, 3%) compared to the DES group (0, 0%; *p* = 0.04). Neither probable nor definite stent thrombosis was observed.

**Figure 2:**
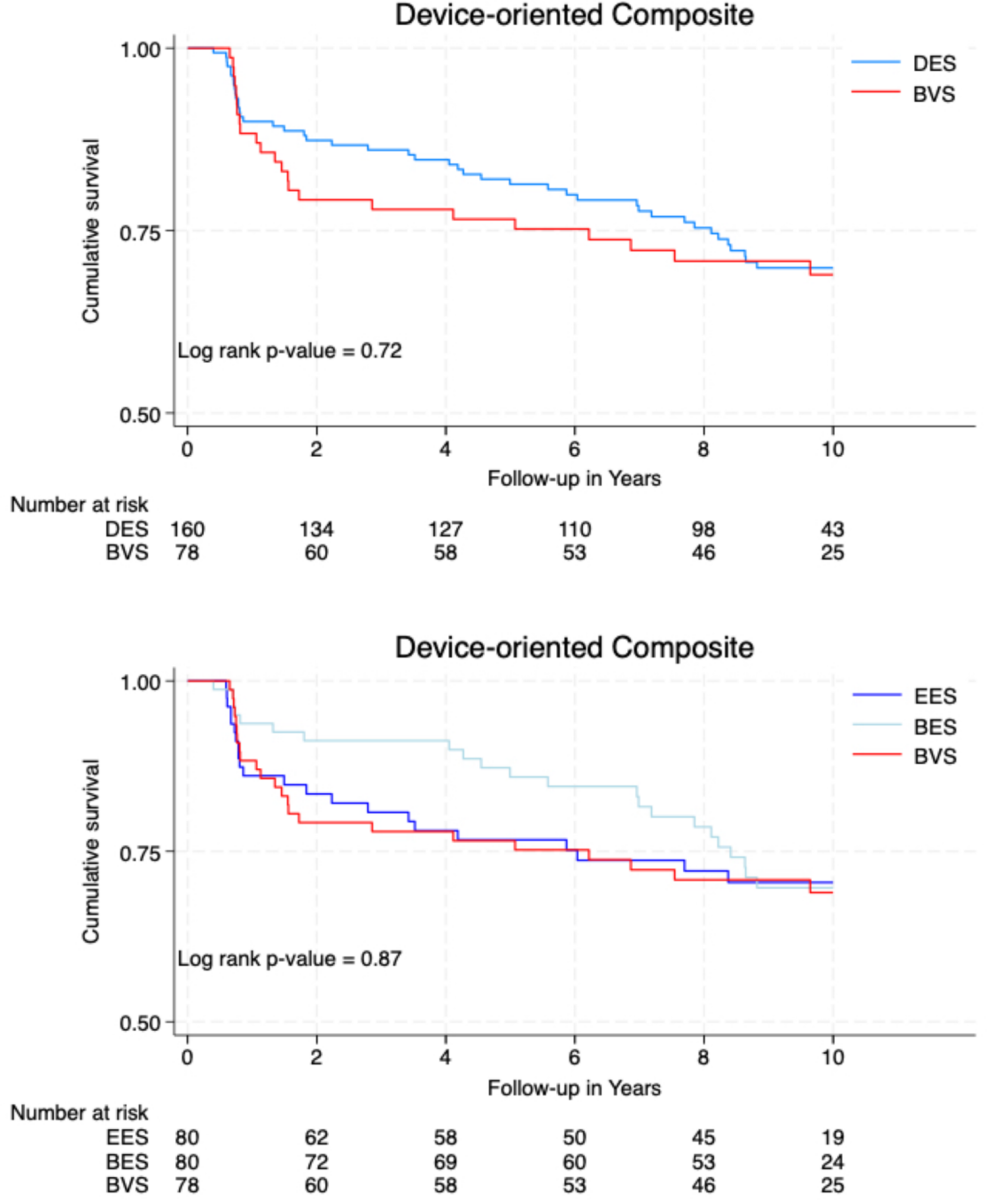
Event-free survival curves.

Figure 2 illustrates the cumulative survival free from the occurrence of DOCEs and POCEs according to the type of implanted stent/scaffold.

**Figure:**
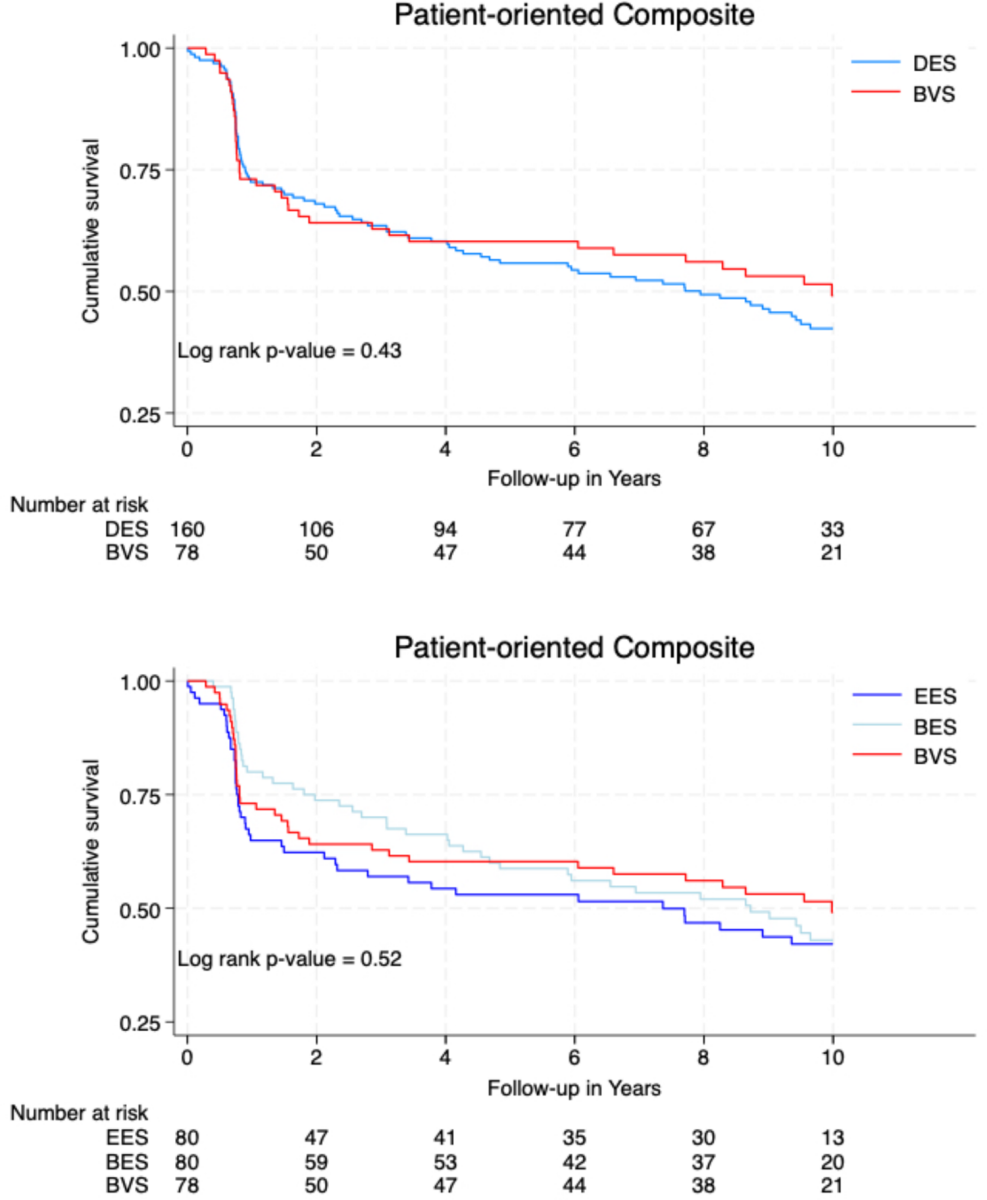


## Discussion

In the EverBio-2 trial, we compared a bioresorbable vascular scaffold with everolimus- and biolimus-eluting metallic stents in routine PCI. Initial analysis revealed comparable clinical outcomes for DESs and BVSs at 9 months (19), followed by subsequent studies showing no difference in major clinical outcomes at 2 (20) and 5 years (21) after inclusion in the same population. To our knowledge, this study represents the first examination of clinical outcomes in a head-to-head comparison of BVSs and DESs beyond 5 years.

### Composite endpoints (DOCE and POCE)

This 10-year analysis comparing the Absorb BVS to DESs (EESs and BESs) did not show a significant difference in the major clinical outcomes, DOCEs and POCEs. DOCE occurrences were well balanced in the different groups with 28% in the DES group and 29% in the BVS group. POCE occurrences were insignificantly lower with a BVS (49%) than a DES (55%) (*p* = 0.43). Comparatively, in the study of the same population at 5 years, DOCEs were insignificantly more frequent in the BVS group (22%) than in the BES group (14%), but quite similar compared to the DES group (18%). POCE occurrences were insignificantly lower with a BVS (40%) than with a DES (43%). In major BVS studies [ABSORB-JAPAN (23), ABSORB-III (24), ABSORB-IV (25)], at 5 years, DOCEs or target vessel failure (TVF) in the BVS groups ranged from 16.1% to 23.2%, while POCEs ranged from 25% to 29.9%. The number of DOCEs and POCEs in our study showed a certain progression explained by the aging of a polymorbid population.

In the Reset study (26), a randomized trial comparing a new-generation everolimus-eluting stent to a first-generation sirolimus-eluting stent by Shiomi et al., at 10 years, among the 1,446 patients who received an everolimus-eluting stent, 26.7% experienced a TVF with 19.6% target lesion failure (TLF). These results align well with what we observed as investigators.

It is important to note that the significant increase in DOCEs and POCEs observed in our study is likely influenced by the high rate of revascularization resulting from the two serial angiographic follow-ups at 9 months and 5 years. In addition, it appears that a substantial number of events occurred around 8 years, coinciding with the COVID-19 pandemic (27), which has been associated with chest pain and elevated troponin levels, leading to more unplanned, repeat coronary angiograms. Moreover, multiple deaths of unknown cause, classified as cardiac deaths according to the standardized definitions for clinical endpoints in coronary stent trials, may have further contributed to the increase in DOCEs.

### Risk of myocardial infarction

Although representing a low number of events, the individual endpoint of MI of the target vessel was significantly higher in the BVS group compared to the BES group, as was all-cause MI. This confirms what we had already described at the 5-year follow-up and is consistent with the 5-year results of the ABSORB-JAPAN, ABSORB-III, and ABSORB-IV studies. In the Reset study, at 10 years, among the 1,446 patients who received an everolimus-eluting stent, MI from any vessel was experienced by 8.6% of the patients which is similar to our findings.

### Stent thrombosis

At 10 years, a possible stent thrombosis occurred only in the BVS group (*n* = 2, 3%), representing a significant difference compared to the DES group (*n* = 0, 0%; *p* = 0.04). In the same population at 5 years, there was one occurrence of possible stent thrombosis (1%) in the BVS group, with none in the DES group, which did not represent a significant difference. In major BVS studies (ABSORB-JAPAN, ABSORB-III, and ABSORB-IV), the 5-year stent thrombosis rate ranged from 1% to 3.8% in the BVS groups and this was significantly higher than in those with an EES only in ABSORB-III (BVS = 2.5%, EES = 1.1%; *p* = 0.03).

In the Reset study, at 10 years, among the 1,446 patients who received an everolimus-eluting stent, stent thrombosis was probable or definite in 1.3%.

### Limitations

The present study has some limitations. First, with 240 patients and a follow-up loss of 16 individuals (7%), the sample size is insufficiently powered to detect small differences in clinical outcomes and does not allow for adjustment for multiple comparisons, which could lead to type 1 errors. Second, at the time of inclusion, there was no BVS-specific implantation protocol, resulting in procedural heterogeneity and lower rates of intracoronary imaging, pre-dilatation, sizing, and post-dilatation. This variability may have influenced clinical outcomes differently compared to more recent large-scale trials with dedicated implantation protocols. Finally, this was a single-center trial with uniform procedural strategies among operators, which may limit the generalizability of the findings to other centers.

## Conclusions

**Figure 3:**
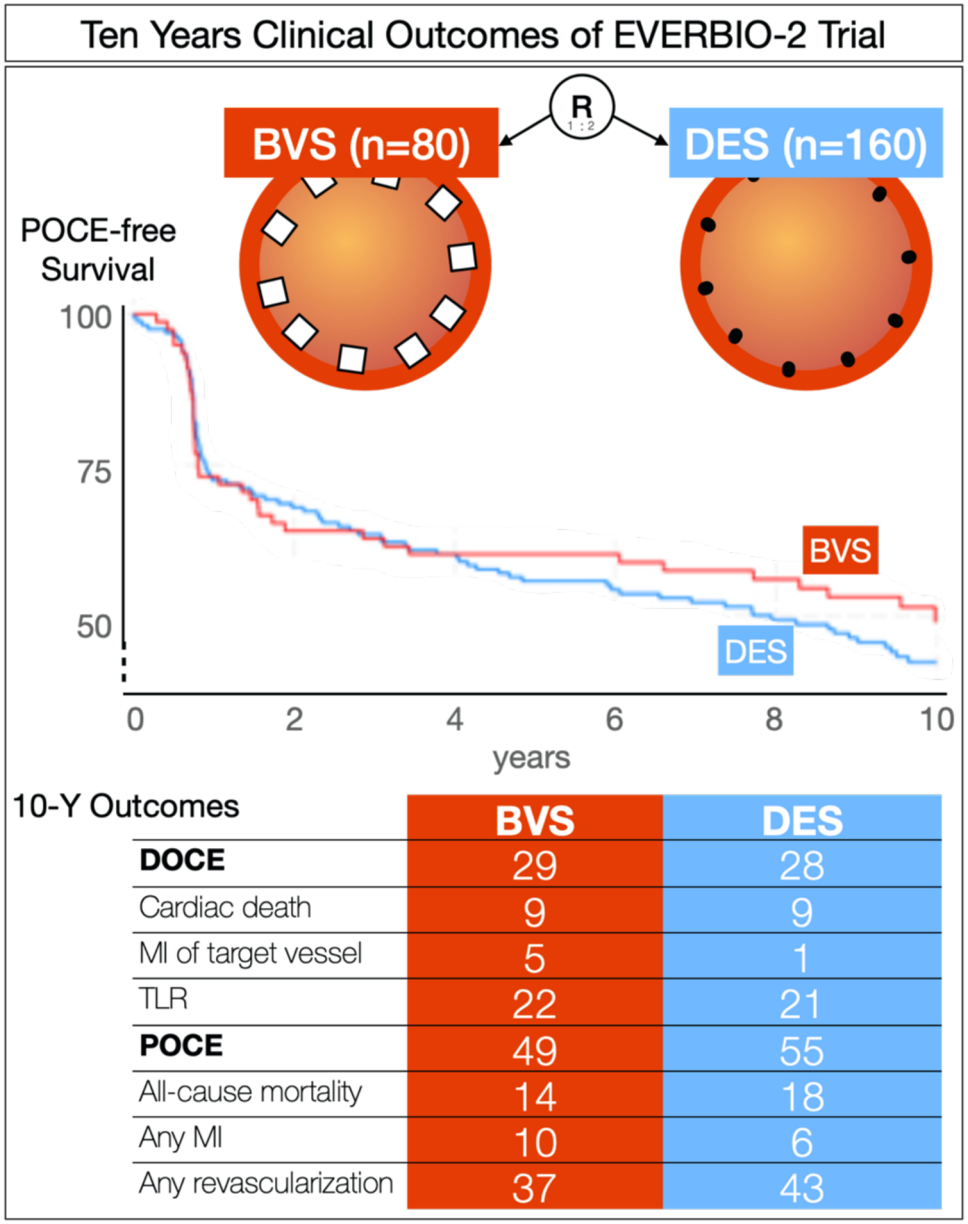
Central illustration.

In this 10-year clinical comparison of BVSs to DESs, we showed no difference in the major clinical outcomes of device-oriented composite or patient-oriented composite endpoints. We found increased rates of target-vessel MI (5%) and all-cause MI (10%) in the BVS group in comparison to BES but not in comparison to DES. The scaffold thrombosis rate was increased in the BVS group (3%) in comparison to the DES group despite low event occurrences (Figure 3).

As the first study available assessing the 10-year clinical evolution of patients who received a bioresorbable drug-eluting vascular scaffold, our clinical investigation shows relative stability in the occurrence of DOCEs and POCEs beyond 5 years without worrying signs indicating a high incidence increase of late stent complications.

## Data Availability

The original contributions presented in the study are included in the article/Supplementary Material, further inquiries can be directed to the corresponding author.
